# A Test of Clairvoyance

**Published:** 1888-10

**Authors:** 


### A TEST OF CLAIRVOYANCE.

Mr. H. G. Moulton, doing business in this city (Boston) on Bulfinch street, recently had an experience in clairvoyance which, as reported in the *Globe* of September 5th, gave him an inkling that there is something more in it than he has hitherto supposed. Frequently, of late, he has missed goods and occasionally a small sum of money, and a fortnight since a considerable sum was taken from the pocket of his vest in a closet. He mentioned this to a young lady in his employ.

A few years ago the young lady's mother lost a small sum of money, which she found from information given her by a clairvoyant medium, and she felt that she could by the same means be of assistance to Mr. Moulton. She obtained a lock of his hair and made a call on the medium. After informing her of what she wanted, the medium passed under control of an Indian spirit, who fully described the store and the persons employed in it, also two boys—one of whom was employed there, the other not—as the guilty ones.

The next morning Mr. Moulton was told of what the medium had said. He was rather skeptical of there being any truth in it, but finally had the boys arrested by an officer, who, in a quiet talk with them, obtained a confession of guilt, and it proved that the one who had committed the theft—the other being only slightly implicated—had been pilfering for a long time, and had put one hundred and twenty dollars in the savings bank. The *Globe* reports the young lady as saying that the medium told the exact amount of money that was missing, and gave the denomination of the bills.—*Banner of Light.*

